# Sudden death complicating a coronary arteritis: polyarteritis nodosa (case report)

**DOI:** 10.11604/pamj.2021.38.113.27601

**Published:** 2021-02-03

**Authors:** Mayssem Gabsi, Sarra Chenik, Houaida Mahfoudhi, Karima Taamallah, Nadhem Hajlaoui, Wafa Fehri

**Affiliations:** 1 Cardiology Department, Military Hospital of Tunis, Tunis, Tunisia

**Keywords:** Coronary artery aneurysm, polyarteritis nodosa, myocardial infarction, sudden death, case report

## Abstract

Coronary artery aneurysms are uncommon, are usually associated with atherosclerosis, and rarely involve all three major coronary arteries. Data on the optimal choice of acute myocardial infarction (AMI)´s revascularization in the context of polyarteritis nodosa (PAN) is limited to case reports and is still an open question. The present report describes a rare case of a young male patient followed for PAN presenting with acute myocardial infarction (AMI). Coronary angiography revealed multiple severe aneurysmal and stenotic changes. Based on clinical feature and angiographic findings, it was strongly suspected that the AMI was a complication of his vasculitis. This case indicates that coronary artery involvement should be carefully monitored during the chronic phase of PAN. The pathophysiology of AMI in PAN patients should be kept in mind and the interventional approach must be performed according to the angiographic findings to avoid complications.

## Introduction

Coronary artery aneurysm is an uncommon disease. The incidence ranges from 0.3% to 4.9% in angiographic studies [1]. A coronary artery aneurysm is characterized by abnormal dilatation of a localized or diffuse segment of the coronary artery tree. The most common cause of coronary artery aneurysm is atherosclerosis. Non atherosclerotic causes are not common. Inflammatory and connective tissue disorders account for 10-20% and include Kawasaki disease, Takayasu arteritis, polyarteritis nodosa (PAN), systemic lupus erythematosus and Ehlers-Danlos syndrome. Other reported causes include infectious diseases such as Lyme borreliosis, septic emboli, and syphilis. The remaining causes are congenital and iatrogenic. The present report describes a rare case involving a young male who presented with acute myocardial infarction (AMI) due to coronary artery occlusion related to multiple huge aneurysms and stenotic lesions in PAN.

## Patient and observation

A 45-year-old male presented two days after an accident of the public way with acute severe chest pain lasting for more than 30 minutes at rest. A pulmonary embolism has been eliminated. He had no history of diabetes mellitus or smoking. However, he is followed for polyarteritis nodosa since 2012 and has as antecedents a cerebral vascular accident (CVA) in 2017. He had no family history of any heart disease. He denied having had any febrile disease or weight loss within the previous several months. Physical examination revealed a hemiparesis of the right hemicorps as a sequela of his CVA, no neuromuscular weakness or skin abnormalities. His vital signs were normal. His cardiovascular exam was unremarkable with a regular rhythm, normal heart sounds, and no murmurs. The chest was clear on auscultation and limbs were warm without any edema. An electrocardiogram on admission revealed a sinus rhythm and sequelae of necrosis in the territories inferolaterobasal. Serum cardiac enzyme levels showed a rise in troponin-T to 30299ng/L. Transthoracic echocardiography revealed severe hypokinesia in the anterior, anterolateral, inferolateral and inferior segments of the left ventricle (LV).

**Figure 1:**
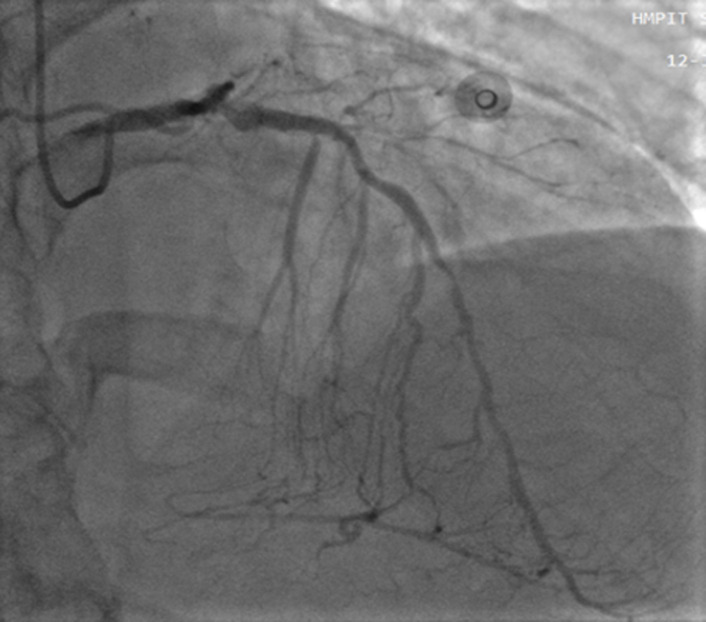
right anterior oblique cranial view shows minimal aneurysmal dilatation in the LAD artery

**Figure 2:**
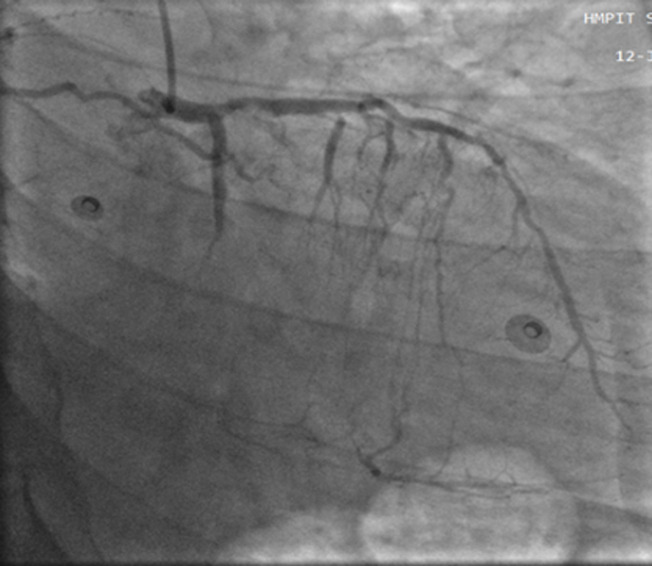
right anterior oblique caudal showing critical stenotic lesion of the proximal segment in the left anterior descending artery and a totally occluded Cx artery

**Figure 3:**
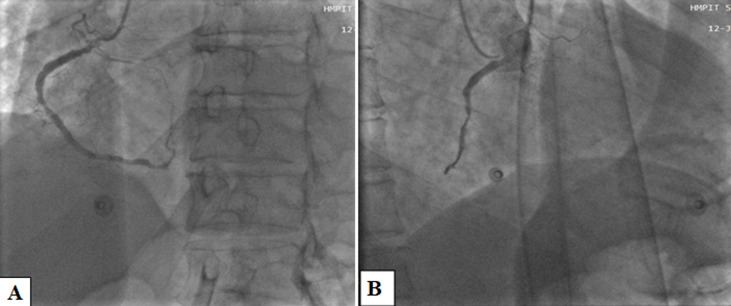
A, B) left anterior oblique cranial and right anterior oblique views shows minimal aneurysmal dilatation and critical stenotic lesion of the mid segment in the right coronary artery

Under the impression that the patient had had an AMI, he was initially treated with standard medical therapy, including acetylsalicylic acid 100 mg/d, clopidogrel 75 mg/d, bisoprolol 2.5 mg/d, ramipril 2.5 mg/d, and intravenous heparin. Coronary angiography revealed huge multiple aneurysmal changes and stenotic lesion involving the left main and all three major coronary arteries (Figure 1). The left circumflex (LCx) artery was totally occluded (Figure 2). Two stenosis were found on the left anterior descending (LAD) artery and we found a stenosis on the right coronary artery (RCA) (Figure 3). The patient was treated with high-dose steroids (prednisolone 60 mg/d) and an immunosuppressive agent (cyclophosphamide 100 mg/d) in addition to the aforementioned standard cardiac treatments and we decided to treat the two axes RCA and LAD. First, a drug-eluted stent was deployed to the mid segment in the right coronary artery, then two drug-eluted stents were deployed to the two consecutive lesions at the proximal and mid segments in the left anterior descending artery. No complications were encountered during the procedure and TIMI 3 flow was achieved in the two axes. The patient was followed up in the coronary care unit. Thereafter, he presented sudden cardiac arrest probably related with acute stent thrombosis.

## Discussion

This case report describes a young man patient who presented with AMI and showed multiple aneurysmal deformations as well as stenotic and occlusive lesions in the coronary arteries which constitutes a very challenging therapeutic management. Although coronary atherosclerosis is the most common cause, many non-atherosclerotic processes can lead to this imbalance by either decreasing coronary blood flow or increasing myocardial metabolic demand. These non-atherosclerotic coronary artery diseases (non-ASCD) include coronary embolism, vasculitis, dissection, vasospasm, and congenital coronary artery anomalies. In addition, systemic non primary cardiac diseases such as pheochromocytoma, thyrotoxicosis, and tetanus can increase metabolic needs of myocardium with resultant relative ischemia despite normal blood flow.

Schrader *et al*. reviewed the clinical and pathologic findings in 36 autopsied patients with polyarteritis nodosa at Johns Hopkins University between 1935 and 1976 to characterize the cardiac involvement in this disease. They found evidence of active or healed coronary arteritis in 18 (50%). The most typical severe lesions involved small subepicardial vessels just as they enter the myocardium rather than deeper intramyocardial arteries. These were characterized by lymphocytic infiltration of the media and adventitia [2]. The more advanced lesions had necrosis and infiltration of the full thickness of the vessel wall with involvement of the surrounding perivascular connective tissue. Thrombosed vessels were occasionally seen. Classic PAN may involve almost any organ system. Involvement of a particular organ system includes: renal failure or hypertension in renal involvement; peripheral mono- or polyneuropathy in peripheral nervous system involvement; exercise-induced angina, AMI, or congestive heart failure in cardiac involvement; and abdominal pain, nausea, vomiting, or gastrointestinal (GI) hemorrhage in GI involvement [3].

PAN is a systemic necrotizing vasculitis that mainly involves small and medium sized arteries. Secondary changes in involved arteries are common and include characteristic aneurysmal formation, intravascular thrombosis, and segmental luminal narrowing. The evolution of multiple vascular aneurysms was the most typical angiographic finding of PAN. Multiple aneurysms are identified in 60-80% of patients, but they can be encountered in other forms of vasculitis [4]. The proximal and middle segments of the RCA are the most common sites for coronary artery aneurysm, followed by the proximal LAD and LCx arteries. Involvement of all three major coronary arteries or the left main stem is very rare, which is the case of our young patient. Although the evolution of multiple aneurysms is the most typical coronary angiographic finding for PAN, rare cases of coronary dissections have been reported. Vasculitis involving the coronary arteries can lead to vessel occlusion and MI. Autopsy series regarding PAN frequently reported evidence of MI with both acute and healing lesions [5]. The most common cardiac manifestation of PAN is heart failure; however, there are occasionally reports of patients presenting with acute MI [6] which is the case of our young patient.

Cardiac involvement in patients suffering from PAN was demonstrated previously and particularly congestive heart failure was reported as a relatively frequent complication. The pathogenesis of heart failure in PAN patients is multifactorial and not yet entirely clarified. Active or healed vasculitis of the coronary arteries as well as renovascular hypertension were identified as major determinants. Nevertheless, the occurrence of manifest AMI is extremely rare in PAN patient. In previous reports, PAN-related AMI was found particularly in patients displaying severe coronary artery affections including multiple aneurysms such as our patient´s case. Coronary aneurysm formation is associated with PAN. However, aneurysms have also been reported with systemic lupus erythematosus (SLE), thrombotic thrombocytopenic purpura (TTP), Kawasaki´s syndrome, atherosclerosis, muscular dysplasia, Takayasu´s arteritis, and Wegener´s granulomatosis. Of these SLE and Takayasu´s are rarely associated with aneurysm formation. Generally, these aneurysms are large and proximal and are in the setting of other obvious clinical markers of systemic disease. Clinical signs may include serositis, hematologic abnormalities, renal disease, dermatologic findings, abdominal angina, and neurologic findings such as weakness, central nervous system vasculitis, and radiculopathy.

Regarding our patient, there were multiple stenoses and minimal aneurysmal dilatation in the LAD and RCA arteries and it was challenging to decide of how we will treat him. There is still no published statement regarding the treatment of coronary stenosis related to PAN. Because of the clinical presentation with AMI and ongoing chest pain, it seems rational to promptly perform coronary angiography and also angioplasty in these patients. In some centers they did not perform a percutaneous coronary intervention. Instead, they treated patients medically with standard cardiac treatments. In addition, they give aggressive anti-inflammatory therapy with high-dose steroids and cyclophosphamide. We report a similar case of a patient who was treated medically because myocardial SPECT showed only minimally reversible ischemia in the LCx area and the patient did not complain of recurrent chest pain. He did not develop further chest pain and remained event-free.

On the other hand, in other centers, patients are referred for surgical revascularization. Data on the optimal choice of bypass grafting in the context of PAN is limited to case reports and is still an open question [7]. Insights regarding graft choice in PAN can be gleaned from surgical revascularization in more common forms of arteritis, such as Kawasaki and Takayasu diseases. Kawasaki disease is a medium-vessel arteritis often seen in the pediatric population. In 156 adult patients with Kawasaki disease who underwent coronary artery bypass grafting (CABG). Left internal mammary artery (LIMA) is preferred, and patency is 91% at 15 years, comparable with all CABG patients. Thus far, 10 cases of mammary artery involvement of PAN have been described, interestingly all in women [8]. This author believes that the demonstrated long-term patency with the use of the internal mammary artery bypass graft outweighs the potential risk of arteritis development. However, angiographic imaging of the LIMA to rule out stenosis or aneurysm development may be appropriate. Between radial artery and saphenous vein grafts (SVG), given their comparative patency rates, we believe that the use of the SVG to avoid the potential complication of arterial involvement is reasonable. Of note, pedicled SVGs are used routinely at our institution on the basis of demonstrated greater short-term patency [9].

Regarding our case, we did perform percutaneous coronary intervention by stenting RCA and LAD. On the other hand, internists have increased the corticosteroid´s dose and introduced the azathioprine. This case indicates that coronary artery involvement should be carefully monitored during the chronic phase of PAN. The pathophysiology of AMI in PAN patients should be kept in mind and the interventional approach must be performed according to the angiographic findings to avoid complications.

## Conclusion

The increased diagnosis rates of AMI in patients under 45 years of age with PAN are most striking, with up to 268.1 events per 10,000 patient years, over 30 times higher than individuals without vasculitis [10]. The cause of such an elevation may be multifactorial. Note that for PAN complicated by multiple coronary artery aneurysm presenting with myocardial infarction, there are no published guidelines for coronary angiography or angioplasty. Our patient sudden death was most likely from arrythmias following the occlusion of his coronary artery. Data on the optimal choice of AMI´s revascularization in the context of PAN is limited to case reports and is still an open question. Further long-term follow-up is required to determine the optimal treatment in this patient population.
